# Synthesis of Novel Quaternary Ammonium Salts and Their *in Vitro* Antileishmanial Activity and U-937 Cell Cytotoxicity

**DOI:** 10.3390/molecules21040381

**Published:** 2016-03-29

**Authors:** Sandra M. Duque-Benítez, Luz Amalia Ríos-Vásquez, Rogelio Ocampo-Cardona, David L. Cedeño, Marjorie A. Jones, Iván D. Vélez, Sara M. Robledo

**Affiliations:** 1 Department of Chemistry, Universidad de Caldas, AA275 Manizales, Colombia; samidube@gmail.com (S.M.D.-B.); rogelio.ocampo@ucaldas.edu.co (R.O.-C.); 2 Department of Chemistry, Illinois State University, Normal, 61790 IL, USA; daleceme@gmail.com (D.L.C.); majone3@ilstu.edu (M.A.J.); 3 PECET, Medical Research Institute, School of Medicine, University of Antioquia, 0500100 Medellín, Colombia; ivan.velez@udea.edu.co

**Keywords:** leishmaniasis, halomethylated quaternary ammoniun salts, halomethylated choline analogs, *Leishmania (V) panamensis*, antileishmanial activity, cytotoxicity

## Abstract

This work describes the synthesis of a series of quaternary ammonium salts and the assessment of their *in vitro* antileishmanial activity and cytotoxicity. A preliminary discussion on a structure-activity relationship of the compounds is also included. Three series of quaternary ammonium salts were prepared: (i) halomethylated quaternary ammonium salts (series I); (ii) non-halogenated quaternary ammonium salts (series II) and (iii) halomethylated choline analogs (series III). Assessments of their *in vitro* cytotoxicity in human promonocytic cells U-937 and antileishmanial activity in axenic amastigotes of *L. (Viannia) panamensis* (M/HOM/87/UA140-pIR-eGFP) were carried out using the MTT (3-(4,5-dimethylthiazol-2-yl)-2,5-diphenyl-tetrazolium bromide) micromethod. Antileishmanial activity was also tested in intracellular amastigotes of *L. (V) panamensis* using flow cytometry. High toxicity for human U937 cells was found with most of the compounds, which exhibited Lethal Concentration 50 (LC_50_) values in the range of 9 to 46 μg/mL. Most of the compounds evidenced antileishmanial activity. In axenic amastigotes, the antileishmanial activity varied from 14 to 57 μg/mL, while in intracellular amastigotes their activity varied from 17 to 50 μg/mL. *N*-Chloromethyl-*N*,*N*-dimethyl-*N*-(4,4-diphenylbut-3-en-1-yl)ammonium iodide (**1a**), *N*-iodomethyl-*N*,*N*-dimethyl-*N*-(4,4-diphenylbut-3-en-1-yl)ammonium iodide (**2a**), *N*,*N*,*N*-trimethyl-*N*-(4,4-diphenylbut-3-en-1-yl)ammonium iodide (**3a**) and *N*,*N*,*N*-trimethyl-*N*-(5,5-diphenylpent-4-en-1-yl)ammonium iodide (**3b**) turned out to be the most active compounds against intracellular amastigotes of *L. (V) panamensis*, with EC_50_ values varying between 24.7 for compound **3b** and 38.4 μg/mL for compound **1a**. Thus, these compounds represents new “hits” in the development of leishmanicidal drugs.

## 1. Introduction

Leishmaniasis is an infectious disease caused by trypanosomatid parasites of the *Leishmania* genus. At least 20 *Leishmania* species are known to cause different clinical forms of leishmaniasis in humans and they are present in different locations worldwide [1]. There are three main clinical manifestations: visceral (VL), mucocutaneous (ML), and cutaneous leishmaniasis (CL), respectively. These vary according to the *Leishmania* species, the geographical region, and the host´s immune response. Leishmaniases are endemic in 98 countries over five continents, with CL being the most prevalent around the world. It is estimated that 12 million people are infected, with 0.7 to 1.2 million new cases occurring every year and 350 million people at risk of infection [2]. The disease disproportionately affects economically and socially disadvantaged people and causes psychological suffering due to the social stigma associated to it [3]. Considering that an effective vaccine is not yet available to protect against leishmaniasis [4], most current research aims at finding alternative treatments of the illness. This has been focused on the development of improved chemotherapies because current medications are not fully satisfactory. Pentavalent antimonials, such as meglumine antimoniate (MA) or sodium stibogluconate are the most widely used antileishmanial drugs, while other medications including pentamidine isothionate, miltefosine, and amphotericin B (AmB) are also available. Although these are reasonably effective, they have serious side effects and require prolonged treatments. Additionally, *Leishmania* parasites are increasingly developing resistance. Altogether, these drawbacks are affecting the adherence of patients to treatments [5]. On the other hand, pharmaceutical companies have demonstrated little motivation to invest in the generation and production of new treatments, thus, since 2002 leishmaniasis has been considered a neglected disease by the World Health Organization [6]. Therefore, academic centers have taken the challenge to investigate and develop therapies that are more accessible, safer, easier to administer, while more effective at low dose with short-length treatments, and at a reasonable cost.

It is well known that certain quaternary ammonium salts are widely used by the pharmaceutical industry for their recognized activity against fungi, bacteria, viruses and parasites. For example, there are reports on their antimicrobial activity against *Cryptococcus neoformans*, *Candida albicans*, *Saccharomyces cerevisiae*, *Aspergillus fumigatus*, *A. niger*, *Mucor mucedo* [7,8]. Human immunodeficiency virus-1 [9], *Staphylococcus aureus*, *Streptococcus faecalis*, *Escherichia coli*, *Proteus vulgaris* [8], *Plasmodium falciparum* [10], *L. donovani* and *L. infantum* [11,12].

Miltefosine (hexadecylphosphocholine), a particular example of a quaternary ammonium salt bearing an apolar C16 chain and a phosphoester group, was initially used as antitumor drug and subsequently was approved for the oral treatment of both cutaneous and visceral leishmaniasis [13,14]. Its biochemical mechanism of action, however, is only partially known. It is generally accepted that the biosynthesis of phosphatidylcholine in *Leishmania* spp. parasites is strongly dependent on choline [15]; thus, quaternary ammonium salts, acting as choline structural analogs, might reduce the content of phosphatidylcholine and phosphatidyl ethanolamine of the parasites, metabolites that are important to synthesize and preserve the structure of the lipid membranes of eukaryotic cells [16].

**Figure 1:**
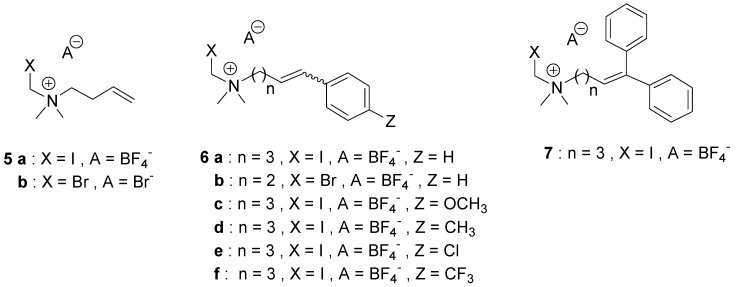
Compounds screened for *in vitro* efficacy against *Leishmania tarentolae* parasites.

Based on the fact that the biosynthesis of phosphatidylcholine in *Leishmania* spp. parasites is strongly dependent on choline [15], and on the *in vitro* and *in vivo* antileishmanial activity demonstrated by quaternary ammonium salts such as edelfosine, ilmofosine and miltefosine [17], a series of *N*-halomethylated quaternary ammonium salts with C4 or C5 side chains bearing monophenyl- and biphenyl-substituted double bonds (Figure 1) were screened *in vitro* as potential antileishmanial agents. The screening revealed that these compounds are active against promastigotes of *L. tarentolae* (unpublished data). Although *L. tarentolae* is a *Leishmania* species that affects certain reptiles but does not infect humans, it has been demonstrated that *L. tarentolae* offer a suitable model during the *in vitro* screening of compounds for activity against *Leishmania* parasite and the identification of compounds with potential as antileishmanial drugs [18].

In this work, various series of quaternary ammonium salts were prepared with the purpose to advance in the identification of “hit” and “lead” compounds as potential candidates for new antileishmanial agents, and trying to establish a possible structure-activity relationship on leishmanicidal activity, especially the effect of the chain length and the presence or absence of a halogen in place of one of the hydrogens of the *N*-methyl. Thus, three series of quaternary ammonium salts were prepared: (i) halomethylated quaternary ammonium salts **1** and **2** (series I), (ii) non-halogenated quaternary ammonium salts **3** (series II) and (iii) halomethylated choline analogs **4a** and **4b** (series III). The assessment of their *in vitro* cytotoxicity in the human promonocytic U-937 cell line and their antileishmanial activity in axenic and intracellular amastigotes of *L. (V) panamensis* was performed, taking into account that this parasite is one of the most pathogenic species for humans in the Americas.

## 2. Results

### 2.1. Synthesis

**Scheme 1:**
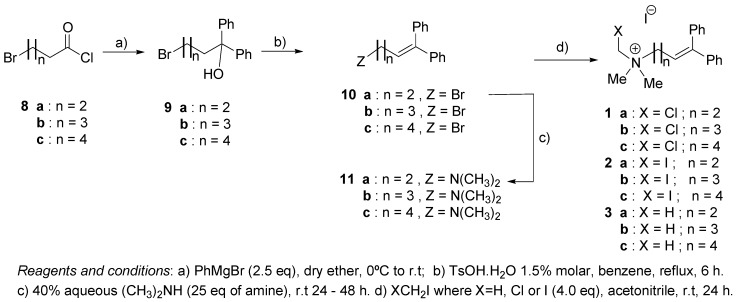
Synthesis of quaternary ammonium salts **1**, **2** and **3** (series I and II).

**Scheme 2:**
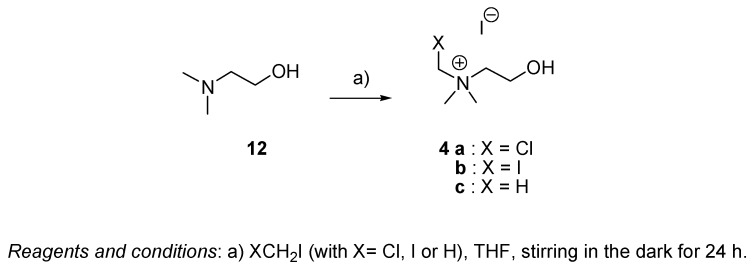
Synthesis of choline **4c** and choline analogs **4a** and **4b**.

Eleven compounds (**1**, **2**, **3**, **4a** and **4b**) and choline **4c** were prepared (Scheme 1 and Scheme 2). Eight of them are new: *N*-chloromethyl-*N*,*N*-dimethyl-*N*-(4,4-diphenyl-3-buten-1-yl)ammonium iodide (**1a**), *N*-chloromethyl-*N*,*N*-dimethyl-*N*-(5,5-diphenyl-4-penten-1-yl)ammonium iodide (**1b**), *N*-chloro-methyl-*N*,*N*-dimethyl-*N*-(6,6-diphenyl-5-hexen-1-yl)ammonium iodide (**1c**) *N*-iodomethyl-*N*,*N*-dimethyl-*N*-(4,4-diphenyl-3-buten-1-yl)ammonium iodide (**2a**), *N*-iodomethyl-*N*,*N*-dimethyl-*N*-(6,6-diphenyl-5-hexen-1-yl)ammonium iodide (**2c**), *N*,*N*,*N*-trimethyl-*N*-(4,4-diphenyl-3-buten-1-yl)-ammonium iodide (**3a**), *N*,*N*,*N*-trimethyl-*N*-(5,5-diphenyl-4-penten-1-yl)ammonium iodide (**3b**) and *N*,*N*,*N*-trimethyl-*N*-(6,6-diphenyl-5-hexen-1-yl)ammonium iodide (**3c**). The other three: *N*-iodo-methyl-*N*,*N*-dimethyl-*N*-(5,5-diphenyl-4-penten-1-yl)ammonium iodide (**2b**), *N*-chloromethyl-*N*-(2-hydroxyethyl)-*N*,*N*-(dimethyl)ammonium iodide (**4a**) and *N*-(2-hydroxyethyl)-*N*-iodomethyl-*N*,*N*-dimethyl)ammonium iodide (**4b**), are known compounds.

Compounds **1a**, **1b**, **1c**, **2a**, **2b**, **2c**, **3a**, **3b** and **3c** were prepared according to the sequence shown in Scheme 1, following or adapting literature methods [18,19] from ω-bromoacyl chlorides **8** by a four-step sequence involving: (i) a Grignard reaction to obtain ω-bromo-α,α-diphenyl alcohols **9** with yields ranging from 68% to 78%; (ii) an acid-catalized dehydration of alcohols **9** using *p*-toluenesulfonic acid in benzene to form the corresponding ω-bromo-α,α-diphenyl olefins **10** with yields beteween 82% and 90%; (iii) a nucleophilic displacement of bromide from the ω-bromo-α,α-diphenyl olefins **10** by dimethylamine to produce ω-(*N*,*N*-dimethylamino)-α,α-diphenyl olefins **11** with yields ranging from 50% to 68%; (iv) a nucleophilic displacement of iodide from iodomethane, chloroiodomethane or diiodomethane by the ω-(*N*,*N*-dimethylamino)-α,α-diphenyl olefins **11** to obtain the target quaternary ammonium salts (series I and II) with yields between 61% and 85%. Choline **4c** and choline analogues **4a** and **4b** were prepared as shown in Scheme 2, following the method previously reported [20]. The halomethylated cholines iodides **4a** and **4b** were synthesized from *N*,*N*-dimethyl-2-hydroxyethanamine (**12**), which was treated with 1.5 equivalents of the corresponding dihalomethane.

### 2.2. In Vitro Cytotoxicity of Quaternary N-(Halomethylated) Ammonium Salts ***1*** and ***2***, Non N-(Halomethylated) Ammonium Salts ***3*** and Halogenated Cholines ***4***

Compounds were tested first for toxicity on U937 human macrophages at four serial dilution concentrations (200, 50, 12.5 and 3.25 μg/mL) and their LC_50_ values were determined (Table 1). Choline **4c** was also tested as a reference compound. Based on our own classification all of the compounds showed LC_50_ < 100 μg/mL and were therefore classified as highly cytotoxic, with **2a** being the most cytotoxic (LC_50_ = 46 μg/mL). As expected, choline **4c** showed a very low level of cytotoxicity, with LC_50_ = 372 μg/mL. AmB showed high cytotoxicity, while MA was potentially non-cytotoxic to U-937 cells.

Antileishmanial activity of quaternary *N*-(halomethylated) ammonium salts **1** and **2**, non-*N*-(halomethylated) ammonium salts **3** and halogenated cholines **4a** and **4b** was assessed in both intracellular (inside mammalian U-937 cells) and axenic amastigotes of *L. (V) panamensis* and compared to the activity of choline **4c**. All compounds showed antileishmanial activity against both amastigotes (intracellular and axenic) of L. (V) panamensis with EC_50_ values <50 μg/mL, except **1b** that exhibited an EC_50_ = 56.3 μg/mL in axenic amastigotes (Table 1). Compound **2c** showed the highest activity with EC_50_ values of 17.6 μg/mL and 14.0 μg/mL in intracellular and axenic amastigotes, respectively. Compounds **2b**, **3b** and **4b** were also active against both types of amastigotes with EC_50_ values of 24.0 μg/mL, 24.7 μg/mL and 23.9 μg/mL, respectively (in intracellular amastigotes) and 19.7 μg/mL, 25.0 μg/mL and 19.6 μg/mL, respectively (in axenic amastigotes) (Table 1). Activity of compounds **3a** and **4a** was high in axenic amastigotes (EC_50_ 25.0 and 24.3 μg/mL, respectively) but moderate in intracellular amastigotes (EC_50_ 30.0 and 29.6 μg/mL, respectively). The remaining compounds **1a**–**c**, **2a** and **3c** showed moderate activity with EC_50_ values varying from 27.8 μg/mL to 49.4 μg/mL in intracellular amastigotes and from 34.1 μg/mL to 40.5 μg/mL in axenic amastigotes. As expected, compound **4c** (choline) showed almost no activity against intracellular parasites (EC_50_ = 149.5 μg/mL) and moderate activity against axenic amastigotes (EC_50_ < 50 μg/mL). AmB, one of the used antileishmanial drug, was active against both amastigote types with EC_50_ values <1.0 μg/mL while MA was active only against intracellular amastigotes (EC_50_ = 6.33 μg/mL) and was not active against axenic parasites (Table 1).

## 3. Discussion

Based on the facts that: (i) choline is important to the biosynthesis of phosphatidylcholine in *Leishmania* parasites [15]; (ii) some quaternary ammonium salts such as edelfosine, ilmofosine and miltefosine have antileishmanial activity [17] and; (iii) some quaternary ammonium compounds may efficiently inhibit choline transport in other intracellular parasites such as *P. falciparum* [19], this work reports the synthesis of a series of new quaternary ammonium salts and their *in vitro* effectiveness against the human pathogen *Leishmania (V.) panamensis*, which causes cutaneous and mucocutaneous leishmaniasis.

As shown in Table 1, chlorinated compounds of the series I characterized by the general structure [ClCH_2_N(CH_3_)_3_(CH_2_)nCH=C(Ph)_2_]^+^I^−^ with *n* = 2, 3 and 4 (**1a**, **1b**, and **1c**, respectively) showed moderate to low activity against axenic amastigotes of *L. (V) panamensis*, with EC_50_ values ranging between 35 and 57 μg/mL, and moderate activity against intracellular amastigotes with EC_50_ values between 38 and 50 μg/mL. These compounds also exhibited high cytotoxicity in U-937 cells with LC_50_ values ranging from 21 to 45 μg/mL. Compound **1a** (*n* = 2) was the most active against axenic amastigotes while **1b** (*n* = 3) was less active than **1c** (*n* = 4). Similarly, **1a** (*n* = 2) exhibits the best activity against intracellular amastigotes while **1c** (*n* = 4) is the worst. In contrast, **1a** (*n* = 2) exhibited the lowest cytotoxicity, while **1b** (*n* = 3) and **1c** (*n* = 4) exhibit similar toxicity.

The selectivity index (SI) is the ratio of the toxicity and activity of a compound, thus it provides a measurement of the balancing effect of these opposing effects. Large values of SI indicate that a compound is relatively active and presents a low toxicity. SI values ranging from 0.4 to 1.3 were found for these three compounds related to their activity against axenic amastigotes. Similar SI values were found in relation to their activity against intracellular amastigotes. In both cases, **1a** (*n* = 2) has the best SI. A comparison of compounds **1a**, **1b** and **1c** with respect to chlorocholine **4a** (from series III), reveals that **4a** is more effective against both axenic and intracellular amastigotes (EC_50_ decreases), but the toxicity of **4a** (LC_50_ = 17.9 μg/mL) is more than two times larger than that for **1a** (LC_50_ = 44.4 μg/mL). The results imply that the terminal diphenylethylene moiety in the tail of the *N*-chlorometyl-*N*,*N*-dimethyl quaternary salts moderates both toxicity and activity with respect to chlorocholine **4a**. Thus, when taking both activity and toxicity into account, compound **1a** (*n* = 2) is the *N*-chloromethyl quaternary salt that exhibits the best anti-leishmanial profile in this study. An interesting trend observed for these chlorinated salts is that, upon lengthening the chain, the activity against both axenic amastigotes and intracellular amastigotes decreases but the toxicity steadily increases.

The iodinated compounds **2** of the series I are salts with the structure [ICH_2_N(CH_3_)_3_(CH_2_)nCH=C(Ph)_2_]^+^I^−^ with *n* = 2, 3 and 4 (**2a**, **2b** and **2c** respectively) exhibit high to moderate activity against axenic amastigotes, with EC_50_ values varying between 14.0 and 36.0 μg/mL and high activity against intracellular amastigotes with EC_50_ values ranging between 17.0 and 28.0 μg/mL. Contrary to the chlorinated analog salts 1, the most active *N*-iodomethyl quaternary salt of series I turned out to be the one with the longest aliphatic chain between the quaternary ammonium head and the diphenyl tail, **2c** (*n* = 4), with an EC_50_ value of 14.0 μg/mL against axenic amastigotes and 17.6 μg/mL against intracellular amastigotes. Also, these iodinated salts were found to have high toxicity with LC_50_ values ranging between 9 and 46 μg/mL. Interestingly, a comparative analysis of *N*-chloromethyl and *N*-iodomethyl quaternary salts implies that the chlorine atom exerts an opposite effect with respect to the iodine atom on the activity against both axenic and intracellular amastigotes but a parallel effect on the toxicity in terms of the effect of the length of the tether chain. However, the effect of lengthening the chain on toxicity is more sensitive when an iodine atom rather than a chlorine atom is present in the cationic ammonium head. For instance, upon lengthering the chain from *n* = 2 to *n* = 4, the LC_50_ value goes from 46 μg/mL for **2a** (*n* = 2) to 10.1 μg/mL for **2b** (*n* = 3) and 9.5 μg/mL for **2c** (*n* = 4). In general, iodomethylated salts were both more active against *Leishmania* parasite and toxic to cells. In terms of the balance between toxicity and antileishmanial activity, the SI of the *N*-iodomethyl quaternary salts of series I (**2a**, **2b**, and **2c**) varies from 0.5 to 1.3 for axenic amastigotes and from 0.4 to 1.7 for intracellular amastigotes, with the higher values corresponding to **2a** (*n* = 2). So, **2a** (*n* = 2) would be the most appropriate if we were to choose a lead compound on the basis of its SI value only.

*N*-(2-Hydroxyethyl)-*N*-iodomethyl-*N*,*N*-dimethylammonium iodide (**4b**) of series III (iodocholine for short) was also tested. The EC_50_ for iodocholine **4b** is similar to that determined for **2b** (*n* = 3) against both axenic and intracellular amastigotes. However, **4b** is a slightly less toxic than **2b** and **2c** (LC_50_ = 17.8 μg/mL for **4b**, 10.1 μg/mL for **2b** and 9.5 μg/mL for **2c**), and more toxic than **2a** (LC_50_ = 46 μg/mL).

Although in general the iodomethylated salts of the series I behave similar to iodocholine **4b**, we can observe higher bioactivity of the *N*-iodomethyl quaternary salt bearing a diphenylethylene unit in the tail and a carbon tether of *n* = 4 (*i.e.*, **2c**).

Compounds of the series II, characterized by the general structure [CH_3_N(CH_3_)_2_(CH_2_)nCH=C(Ph)_2_]^+^I^−^ with *n* = 2, 3 and 4 (**3a**, **3b** and **3c** respectively) exhibit high toxicity with the lowest being for **3a** (*n* = 2, LC_50_ = 39.2 μg/mL) while increasing when going to *n* = 3 and *n* = 4, with **3c** (*n* = 4) being the most toxic. These compounds exhibited moderate activity against both axenic and intracellular amastigotes, and it seems that the activity is sensitive to the length of the tether chain in going from *n* = 3 to *n* = 4.

The results clearly indicate that halogen atoms and the length of the tether chain modify the biological profile of the quaternary salts. In general, the toxicity increases upon lengthening the chain for a particular X = H (series II), Cl or I (Series I). Interestingly, for both X = Cl and I, the toxicity largely decreases with the addition of a methylene unit in the tethering chain from *n* = 2 to *n* = 3 but remains approximately the same with the increase of an additional methylene unit to *n* = 4. As previously mentioned, there is a larger decrease when X = I as LC_50_ decreases four-fold, while when X = Cl the LC_50_ is halved. In slight contrast, when X = H the toxicity seems to step-up as an additional methylene unit is added to the tethering chain (LC_50_ decreases about 12–14 μg/mL per additional methylene unit). The changes in the antileishmanial activity are less clear although trends can be discerned as previously noted.

We hypothesize that the molecular structure of the salt (*i.e.*, diphenylethylene moiety and its proximity to the ammonium head) as well as the presence and identity of the halogen in the ammonium head influence the activity and toxicity of *N*-halomethylated quaternary salts. Further knowledge of the interactions between the compounds and proteins or enzymes of the parasite will be key to understanding the relationship between structure and the differential cytotoxicity and anti-leishmanial activity. Indeed, the conformational structure of each compound constitutes a piece of information in the overall picture of the biological behavior of these salts. For that purpose, a recent X-ray diffraction study of the solid state structures of the iodinated compounds of the series I (**2a**, **2b** and **2c**) was carried out [20]. Interestingly, the solid state conformation of **2a** (*n* = 2) differs from that of the **2b** (*n* = 3) and **2c** (*n* = 4). The study revealed that conformations may be influenced by supramolecular interactions as well as intramolecular interactions. The understanding of these interactions will be important in both establishing mechanisms of bioavailability of the compounds as they go from the solid state to a liquid/solution phase and on how the most stable conformation of the compounds interact with enzymes or proteins of the parasite. A patent has been granted for compounds **1a**, **1c**, **2a** and **2c** [21]. This patent is one of the at least 10 patents that have been granted during the last 10 years to various cores and their derivatives that have been reported to possess antileishmanial activity [22].

## 4. Materials and Methods

### 4.1. Structural Characterization

Structural characterization of compounds was carried out by ^1^H-NMR, ^13^C-NMR, elemental analysis and mass spectrometry. ^1^H-NMR and ^13^C-NMR spectra were obtained at 300 MHz, 400, 500 and 126 MHz and 75 MHz respectively on a VXR-300, a Gemini-300 both Varian Medical System (Palo Alto, CA, USA) or a Bruker (Billerica, MA, USA) 500 MHz spectrometer. Elemental analyses of carbon, hydrogen and nitrogen were performed at the microanalysis laboratory at the University of Illinois (Urbana-Champaign, IL, USA). Mass spectra were obtained on a HP Series 1100 MSD ESI-MS (Hewlett Packard, Palo Alto, CA, USA) electrospray ionization/mass spectrometer,) using a solution of 0.1% formic acid in acetonitrile as solvent injection. Samples were dissolved in acetonitrile (4 mg/mL) prior to injection into the instrument. Melting points were determined on a capillary Thomas-Hoover melting point apparatus (Swedesboro, NJ, USA).

### 4.2. Synthesis

#### 4.2.1. General Procedure for the Preparation of ω-Bromo-α,α-diphenyl Alcohols **9**

The methodology used was according to Horner *et al.* [23]. All glassware was flame-dried under an inert atmosphere, and reactions were carried out under argon or dry nitrogen. A commercially available ethereal 3M solution of phenylmagnesium bromide (an appropriate volume for 2.6 equivalents) was poured into a three-necked, round-bottomed flask that was equipped with an addition funnel and a vertical condenser. The solution was cooled with an ice-salt bath and then an ethereal solution of the respective ω-bromoacyl chloride **8** (1 equivalent) was slowly added from the addition funnel with continuous magnetic stirring while the reaction mixture was kept at about 0 °C. Once the addition was finished, the reactant mixture was kept at 0 °C during 15 min and then allowed to slowly reach room temperature. After that, a saturated aqueous NH_4_Cl solution was carefully added and the whole mixture was transferred to a separatory funnel. The organic layer was separated, washed with saturated aqueous NaCl solution and dried with anhydrous MgSO_4_. After filtration, solvent was removed by rotary evaporation under reduced pressure. The products were purified by flash column chromatography (hexane:ethyl acetate 5:1 as eluent). White solids or yellow oils were usually obtained.

*4-Bromo-1,1-diphenylbutanol* (**9a**). Starting from 4-bromobutanoyl chloride **8a** (5.4 mmol), **9a** was obtained as a yellow oil (78% yield). ^1^H-NMR (CDCl_3_, 300 MHz) δ 1.80–1.90 (m, 2H), 2.54–2.94 (m, 2H), 3.33 (t, *J* = 6.9 Hz, 2H), 7.01–7.41 (m, 10H) ppm. ^13^C-NMR (CDCl_3_, 75 MHz) δ 22.92, 30.91, 36.18, 75.34, 123.10–142.24 ppm.

*5-Bromo-1,1-diphenylpentanol* (**9b**). Starting from 5-bromopentanoyl chloride **8b** (5.0 mmol), **9b** was obtained as a white solid (68% yield). ^1^H-NMR (CDCl_3_, 300 MHz) δ 1.53–1.63 (m, 2H), 2.02–2.12 (m, 2H), 2.31–2.41 (m, 2H), 3.47 (t, *J* = 6.9 Hz, 2H), 7.23–7.63 (m, 10H) ppm. ^13^C-NMR (CDCl_3_, 75 MHz) δ 23.12, 28.97, 33.62, 33.97, 41.51, 126.53–143.53 ppm.

*6-Bromo-1,1-diphenylhexanol* (**9c**). Starting from 6-bromohexanoyl chloride **8c** (11.7 mmol), **9c** was obtained as a yellow oil (72% yield). ^1^H-NMR (CDCl_3_, 300 MHz) δ 1.47–1.57 (m, 2H), 1.91–2.01 (m, 2H), 2.03–2.13 (m, 2H), 2.27–2.37 (m, 2H), 3.39–3.49 (m, 2H), 7.17–7.57 (m, 10H) ppm. ^13^C-NMR (CDCl_3_, 75 MHz) δ 23.07, 28.90, 33.53, 33.56, 33.87, 41.46, 126.45–143.48 ppm.

#### 4.2.2. General Procedure for the Preparation of ω-Bromo-α,α-diphenyl Olefins **10**

The methodology was according to Horner *et al.* [18]. A round-bottomed flask was equipped with a Dean-Stark trap and a vertical condenser. Alcohols **9** (**a**, **b** or **c**) and *p*-toluenesulfonic acid monohydrate (*p*-TsOH·H_2_O) in a molar ratio of about 60:1 were dissolved in benzene and refluxed during 6 h. The approximate concentration of the solution was 5 M. After cooling down, the crude mixture was washed with a saturated NaHCO_3_ aqueous solution and then with a saturated NaCl solution. The organic layer was dried with anhydrous MgSO_4_ and filtered, and the solvent was removed by rotary evaporation under reduced pressure. The products were purified by flash column chromatography using hexane as eluent.

*4-Bromo-1,1-diphenyl-1-butene* (**10a**). Starting from **9a** (3.9 mmol), **10a** was obtained as a yellow oil (90% yield). ^1^H-NMR (CDCl_3_, 300 MHz) δ 2.54–2.64 (m, 2H), 3.32 (t, *J* = 6.9 Hz, 2H), 5.99 (t, *J* = 7.2 Hz, 1H), 7.04–7.34 (m, 10H) ppm. ^13^C-NMR (CDCl_3_, 75 MHz) δ 30.57, 30.82, 123.60–142.20 ppm.

*5-Bromo-1,1-diphenyl-l-pentene* (**10b**). Starting from **9b** (3.1 mmol), **10b** was obtained as a yellow oil (82% yield). ^1^H-NMR (CDCl_3_, 300 MHz) δ 1.81–1.97 (m, 2H), 2.08–2.22 (m, 2H), 3.27 (t, *J* = 6.9 Hz, 2H), 5.94 (t, *J* = 7.5 Hz, 1H), 7.01–7.31 (m, 10H) ppm. ^13^C-NMR (CDCl_3_, 75 MHz) δ 26.35, 30.97, 31.23, 124.97–141.23 ppm.

*6-Bromo-1,1-diphenyl-l-hexene* (**10c**). Starting from **9c** (8.2 mmol), **10c** was obtained as a yellow oil (84% yield). ^1^H-NMR (CDCl_3_, 300 MHz) δ 1.66–1.74 (m, 2H), 1.91–2.01 (m, 2H), 2.26 (c, *J* = 7.4 Hz, 2H), 3.47 (t, *J* = 6.9 Hz, 2H), 6.18 (t, *J* = 7.4 Hz, 1H), 7.24–7.54 (m, 10H) ppm. ^13^C-NMR (CDCl_3_, 75 MHz) δ 28.85, 29.26, 32.75, 34.12, 127.42–143.05 ppm.

#### 4.2.3. General Procedure for the Preparation of ω-(*N*,*N*-Dimethylamino)-α,α-diphenyl Olefins **11**

The synthesis of ω-(*N*,*N*-dimethylamino)-α,α-diphenyl olefins **11** was carried out according to the methodology reported by Ríos *et al.* [24]. The reaction of ω-bromo-α,α-diphenyl olefins **10** with a 40% aqueous dimethylamine solution was carried out using the appropriate volume for a molar ratio 26:1 of amine to olefin **10**. The aqueous dimethylamine solution was poured into a round-bottomed flask, and then a tetrahydrofuran (THF) solution of **10**(**a**, **b** or **c**) was slowly added to the amine solution. The mixture was stirred at room temperature overnight and then it was transferred to a separatory funnel and extracted with diethyl ether. The organic layer was separated and extracted with aqueous 10% HCl. The aqueous extract was neutralized with aqueous 10% NaOH and it was extracted again with diethyl ether. The ether extract was dried with anhydrous MgSO_4_ and filtered. The solvent was removed by rotary evaporation under reduced pressure, yielding the expected amines as yellow oils.

*N,N-Dimethyl-4,4-diphenyl-3-butenylamine* (**11a**). Starting from **10a** (3.1 mmol), **11a** was obtained as yellow oil (50% yield). ^1^H-NMR (CDCl_3_, 300 MHz) δ 2.07 (s, 6H), 2.12–2.22 (m, 2H), 2.23–2.33 (m, 2H), 5.97 (t, *J* = 7.3 Hz, 1H), 7.00–7.30 (m, 10H) ppm. ^13^C-NMR (CDCl_3_, 75 MHz) δ 26.14, 43.25, 57.52, 124.76–140.43 ppm.

*N,N-Dimethyl-5,5-diphenyl-4-pentenylamine* (**11b**). Starting from **10b** (2.2 mmol), **11b** was obtained as yellow oil (68% yield). ^1^H-NMR (CDCl_3_, 300 MHz) δ 1.44–1.54 (m, 2H), 2.00–2.10 (m, 2H), 2.14 (s, 6H), 2.21–2.31 (m, 2H), 5.98 (t, *J* = 7.5 Hz, 1H), 7.00–7.30 (m, 10H) ppm. ^13^C-NMR (CDCl_3_, 75 MHz) δ 25.34, 26.26, 43.23, 57.71, 124.28–140.60 ppm.

*N,N-Dimethyl-6,6-diphenyl-5-hexenylamine* (**11c**). Starting from **10c** (6.5 mmol), **11c** was obtained as yellow oil (61% yield). ^1^H-NMR (CDCl_3_, 300 MHz) δ 1.25–1.34 (m, 2H), 1.48–1.57, (m, 2H), 2.18–2.29 (m, 2H), 2.20–2.29 (m, 2H), 2.26 (s, 6H), 6.16 (t, *J* = 7.5 Hz, 1H), 7.20–7.51 (m, 10H) ppm. ^13^C-NMR (CDCl_3_, 75 MHz) δ 27.78, 28.24, 30.06, 45.94, 60.10, 127.21–143.26 ppm.

#### 4.2.4. General Procedure for the Preparation of Quaternary Ammonium Salts **1**–**3** (Series I and II)

Synthesis of series I and II of quaternary ammonium salts was according to the methodology reported by Ríos *et al.* [24]. The reaction of ω-(*N*,*N*-dimethylamino)-α,α-diphenyl olefins **11 a**, **b** or **c**) with a 50% acetonitrile solution of XCH_2_I (with X= Cl or I for the series I, and X = H for the series II) was carried out using a 4:1 molar ratio of XCH_2_I to amine **11**. The starting tertiary amine **11** and the acetonitrile solution of the respective XCH_2_I substrate were mixed in a round-bottomed flask, and the reactions were allowed to run overnight in the dark. The precipitated salts were filtered off and then washed several times with diethyl ether or ethyl acetate, and then recrystallized from water. The pure quaternary ammonium salts were obtained as white solids.

*N-Chloromethyl-N,N-dimethyl-N-(4,4-diphenylbut-3-en-1-yl)ammonium iodide* (**1a**). Starting from **11a** and ClCH_2_I, salt **1a** (85% yield) was obtained as a white solid, m.p. 140–142 °C. ^1^H-NMR (D_2_O, 300 MHz) δ 2.31–2.53 (m, 2H), 3.02 (s, 6H), 3.37–3.55 (m, 2H), 5.11 (s, 2H), 5.99 (t, *J* = 7.3 Hz, 1H), 7.04–7.47 (m, 10H) ppm. ^13^C-NMR (D_2_O, 75 MHz) δ 23.31, 49.42, 61.43, 68.13, 121.97, 127.18–129.46, 138.76, 141.46, 145.22 ppm. Elemental analysis: calc. for C_19_H_23_NICl: C, 53.35%; H, 5.42%; N, 3.27%; found C, 53.35%; H, 5.34%; N, 3.28%. MS-ESI *m*/*z* calc. for [C_19_H_23_NCl]^+^: 300.15 amu, found: 300.20 amu.

*N-Chloromethyl-N,N-dimethyl-N-(5,5-diphenylpent-4-en-1-yl)ammonium iodide* (**1b**). Starting from **11b** and ClCH_2_I, salt **1b** (84% yield) was obtained as a white solid, m.p. 121–124 °C. ^1^H-NMR (DMSO, 300 MHz) δ 1.83–1.93 (m, 2H), 2.06–2.16 (m, 2H), 3.12 (s, 6H), 3.38–3.45 (m, 2H), 5.33 (s, 2H), 6.12 (t, *J* = 7.2 Hz, 1H), 7.07–7.53 (m, 10H) ppm. ^13^C-NMR (DMSO, 75 MHz) δ 24.96, 29.09, 52.01, 64.92, 71.35, 129.93–132.46, 142.13, 144.69, 145.58 ppm. Elemental analysis: calc. for C_20_H_25_NICl: C, 54.37%; H, 5.70%; N, 3.17%; found C, 53.39%; H, 5.57%; N, 3.17%. MS-ESI *m*/*z* calc. for [C_20_H_25_NCl]^+^: 314.17 amu, found: 314.20 amu.

*N-Chloromethyl-N,N-dimethyl-N-(6,6-diphenylhex-5-en-1-yl)ammonium iodide* (**1c**). Starting from **11c** and ClCH_2_I, salt **1c** (61% yield) was obtained as a white solid, m.p. 119–121 °C. ^1^H-NMR (CDCl_3_, 400 MHz) δ 1.53–1.63 (m, 2H), 1.70–1.80 (m, 2H), 2.18–2.26 (m, 2H), 3.46 (s, 6H), 3.60–3.68 (m, 2H), 5.64 (s, 2H), 6.05 (t, *J* = 7.4 Hz, 1H), 7.12–7.42 (m, 10H) ppm. ^13^C-NMR (CDCl_3_, 101 MHz) δ 22.23, 26.25, 28.91, 49.96, 63.15, 68.92, 127.16–129.82, 139.76, 142.25, 142.97 ppm. Elemental analysis: calc. for C_21_H_27_NICl: C, 55.34%; H, 5.97%; N, 3.07%; found C, 55.10%; H, 5.95%; N, 3.10%. MS-ESI *m*/*z* calc. for [C_21_H_27_NCl]^+^: 328.18 amu, found: 328.20 amu.

*N-Iodomethyl-N,N-dimethyl-N-(4,4-diphenylbut-3-en-1-yl)ammonium iodide* (**2a**). Starting from **11a** and CH_2_I_2_, salt **2a** (74% yield) was obtained as a white solid, m.p. 152–154 °C. ^1^H-NMR (DMSO, 300 MHz δ, ppm.): 2.43–2.53 (m, 2H), 3.12 (s, 6H), 3.45–3.55 (m, 2H), 5.05 (s, 2H), 6.07 (t, *J* = 7.4 Hz, 1H), 7.15–7.58 (m, 10H) ppm. ^13^C-NMR (DMSO, 75 MHz, δ, ppm) δ 23.70, 31.49, 51.66, 63.58, 121.92, 127.19–129.51, 138.79, 141.62, 145.03 ppm. Elemental analysis: calculated for C_19_H_23_NI_2_: C, 43.95%; H, 4.46%; N, 2.70%; found, C, 43.48%; H, 4.35%; N, 2.68%. MS-ESI *m*/*z* calculated for cation [C_19_H_23_NI]^+^: 392.09 amu, found: 391.95 amu.

*N-Iodomethyl-N,N-dimethyl-N-(5,5-diphenyl-4-penten-1-yl)ammonium iodide* (**2b**). Starting from **11b** and CH_2_I_2_, salt **2b** (77% yield) was obtained as a white solid, m.p. 157–158 °C. ^1^H-NMR (DMSO, 300 MHz δ, ppm): 1.80–1.90 (m, 2H), 2.07–2.17 (m, 2H), 3.15 (s, 6H), 3.31–3.40 (m, 2H), 5.18 (s, 2H), 6.14 (t, *J* = 7.2 Hz, 1H), 7.11–7.51 (10H). ^13^C-NMR (DMSO, 75 MHz, δ, ppm): 22.30, 25.91, 39.01, 51.19, 63.84, 126.84–141.68. ESI-MS *m*/*z* calc. for [C_20_H_25_NI]^+^: 406.10 amu, found: 406.20 amu.

*N-Iodomethyl-N,N-dimethyl-N-(6,6-diphenylhex-5-en-1-yl)ammonium iodide* (**2c**). Starting from **11c** and CH_2_I_2_, salt **2c** (72% yield) was obtained as a white solid, m.p. 156–158 °C. ^1^H-NMR (DMSO, 300 MHz δ, ppm): 1.42–1.46 (m, 2H), 1.64–1.72 (m, 2H), 2.08–2.15 (m, 2H), 3.10 (s, 6H), 3.33–3.44 (m, 2H), 5.14 (s, 2H), 6.14 (t, *J* = 7.3 Hz, 1H), 7.06–7.51 (m, 10H) ppm. ^13^C-NMR (DMSO, 75 MHz, δ, ppm): 25.07, 28.91, 31.91, 35.35, 54.29, 67.34, 129.89–132.50, 130.19, 142.45, 144.34, 145.02. Elemental analysis: calc. for C_21_H_27_NI_2_: C, 46.09%; H, 4.97%; N, 2.56%; found C, 45.91%; H, 4.93%; N, 2.58%. MS-ESI *m*/*z* calc. for [C_21_H_27_NI]^+^: 420.12 amu, found: 420.20 amu.

*N,N,N-Trimethyl-N-(4,4-diphenylbut-3-en-1-yl)ammonium iodide* (**3a**). Starting from **11a** and CH_3_I, salt **3a** (82% yield) was obtained as a white solid, m.p. 190–192 °C. ^1^H-NMR (DMSO, 300 MHz) δ 2.54–2.63 (m, 2H), 3.11 (s, 9H), 3.53–3.63 (m, 2H), 6.13 (t, *J* = 7.1 Hz, 1H), 7.18–7.66 (m, 10H) ppm. ^13^C-NMR (DMSO 75 MHz,) δ 24.03, 52.52, 64.92, 123.28–144.69 ppm. Elemental analysis: calc. for C_19_H_24_NI: C, 58.02%; H, 6.15%; N, 3.56%; found C, 56.71%; H, 6.23%; N, 3.52%. MS-ESI *m*/*z* calc. for [C_19_H_24_N]^+^: 266.19 amu, found: 266.20 amu.

*N,N,N-Trimethyl-N-(5,5-diphenylpent-4-en-1-yl)ammonium iodide* (**3b**). Starting from **11b** and CH_3_I, salt **3b** (92% yield) was obtained as a white solid, m.p. 196–197 °C. ^1^H-NMR (DMSO, 400 MHz) δ 1.87–1.93 (m, 2H), 2.24 (c, *J* = 7.1 Hz, 2H), 3.37 (s, 9H), 3.43–3.55 (m, 2H), 6.08 (t, *J* = 7.1 Hz, 1H), 7.10–7.45 (m, 10H) ppm. ^13^C-NMR (DMSO, 101 MHz,) δ 23.41, 26.19, 53.32, 66.67, 125.90, 127.14–128.92, 139.39, 141.82, 143.96 ppm. Elemental analysis: calc. for C_20_H_26_NI: C, 58.97%; H, 6.43%; N, 3.44%; found C, 55.93%; H, 6.10%; N, 3.23%. MS-ESI *m*/*z* calc. for [C_20_H_26_N]^+^: 280.21 amu, found: 280.20 amu.

*N,N,N-Trimethyl-N-(6,6-diphenylhex-5-en-1-yl)ammonium iodide* (**3c**). Starting from **11c** and CH_3_I, salt **3c** (72% yield) was obtained as a white solid, m.p. 202–204 °C. ^1^H-NMR (DMSO, 500 MHz) δ 1.40–1.46 (m, 2H), 1.64–1.72 (m, 2H), 2.11 (c, *J* = 7.4 Hz, 2H), 3.03 (s, 9H), 3.20–3.30 (m, 2H), 6.16 (t, *J* = 7.4 Hz, 1H), 7.10–7.49 (m, 10H) ppm. ^13^C-NMR (DMSO, 126 MHz) δ 22.22, 26.22, 29.12, 52.69, 65.82, 127.10–129.12, 129.71, 139.78, 141.76, 142.26 ppm. Elemental analysis: calc. for C_21_H_28_NI: C, 59.86%; H, 6.70%; N, 3.32%; found C, 59.49%; H, 6.74%; N, 3.35%. MS-ESI *m*/*z* calc. for [C_21_H_28_N]^+^: 294.22 amu, found: 294.20 amu.

#### 4.2.5. General Procedure for the Preparation of Choline **4c** and Choline Analogs **4a** and **4b** (Series III)

The compounds **4a**, **4b** and **4c** were prepared following the procedure reported by Mistry *et al.* [25] starting from *N*,*N*-dimethylethanolamine **12** and XCH_2_I in THF (with X = H, Cl or I), using a 1.5:1 molar ratio of XCH_2_I to ethanolamine. The reaction mixture was stirred in the dark for 24 h at room temperature. The gummy crude product was washed with dry THF, and the resulting salts were filtered off to obtain the products as white solids.

*N-Choromethyl-N,N-dimethyl-N-(2-hydroxyet-1-yl)ammonium iodide* (**4a**). Starting from *N*,*N*-dimethyl-ethanolamine (**12**) and ClCH_2_I, **4a** (51.2% yield) was obtained as a white solid, m.p. 101–103 °C ^1^H-NMR (D_2_O, 300 MHz) δ 3.32 (s, 6H), 3.62–3.74 (m, 2H), 4.01–4.10 (m, 2H), 5.28 (s, 2H) ppm. ^13^C-NMR (D_2_O, 75 MHz) δ 33.11, 53.14, 55.95, 66.46 ppm.

*N-Iodomethyl-N,N-dimethyl-N-(2-hydroxyet-1-yl)ammonium iodide* (**4b**). Starting from *N*,*N*-dimethyl-ethanolamine (**12**) and CH_2_I_2_, **4b** (61.5% yield) was obtained as a white solid, m.p. 96–97 °C. ^1^H-NMR (D_2_O, 300 MHz) δ 3.33 (s, 6H), 3.65–3.74 (m, 2H), 4.01–4.13 (m, 2H), 5.29 (s, 2H) ppm. ^13^C-NMR (D_2_O, 75 MHz) δ 33.32, 53.22, 56.14, 66.51 ppm.

*(2-Hydroxyethyl)trimethylammonium iodide (choline)* (**4c**). Starting from *N*,*N*-dimethylethanolamine (**12**) and CH_3_I, **4c** (88% yield) was obtained as a white solid, m.p. 268–270 °C. ^1^H-NMR (D_2_O, 300 MHz), δ 3.20 (s, 9H), 3.49–3.55 (m, 2H), 4.00–4.10 (m, 2H) ppm. ^13^C-NMR (D_2_O; 75 MHz) δ 54.39, 55.63, 67.98 ppm.

The spectral data of the synthesized ω-bromo-α,α-diphenyl alcohols **9**, ω-bromo-α,α-diphenyl olefins **10** and ω-(*N*,*N*-dimethylamino)-α,α-diphenyl olefins **11** were in agreement with those reported previously by Horner *et al.* [18]. All compounds were stored at room temperature until use. Prior to the biological testing, each compound was solubilized in 0.5% dimethyl sulfoxide (DMSO; Sigma-Aldrich, St. Louis, MO, USA), and then diluted to the appropriate concentration in culture media.

### 4.3. Biological Activity

#### 4.3.1. Cells and Culture Conditions

U-937 promonocytes (CRL1593.2™) were obtained from the American Type Culture Collection (ATCC, Manassas, VA, USA), cultured in complete medium containing RPMI 1640 medium (Sigma) supplemented with 10% fetal bovine serum (FBS) (Gibco, Life Technologies, Grand Island, NY, USA) and 1% antibiotics (100 U/mL penicillin and 0.1 mg/mL streptomycin) (Sigma). Cells were maintained in standard conditions at 37 °C, 5% CO_2_ with change of medium every three days until use.

#### 4.3.2. Leishmania Strain and Cultivation of Parasites

Virulent *L. (V) panamensis* MHOM/CO/87/UA140 strain transfected with green fluorescent protein gene called *L. (V) panamensis*-pIR-eGFP was maintained by successive passaging in golden hamsters (*Mesocricetus auratus*). Briefly, amastigotes were aspirated from cutaneous lesions of hamsters experimentally infected with promastigotes of *L. (V) panamensis*-pIR-eGFP. Samples were cultured in biphasic culture with Novy-MacNeal-Nicholle (NNN) medium as solid phase and PBS plus glucose, pH 6.9 as liquid phase and incubated at 26 °C to obtain promastigotes. Medium was changed every week until 2 months.

The axenic amastigotes were obtained from promastigotes cutured in Schneider’s medium, as described elsewhere [26]. Briefly, promastigotes of *L. (V) panamensis*-pIR-eGFP at 5 × 10^6^ parasites/mL were cultured in Schneider medium (Sigma), pH 5.4 supplemented with 20% FBS and 1% antibiotics and incubation at 26 °C. Every three days, medium was changed and temperature was increased one degree each time until it reached 32 °C at which the transformation of the promastigotes into axenic amastigotes is achieved. After three days of growing as axenic amastigotes, parasites were ready to be used in the antileishmanial testing assay as described below.

Intracellular amastigotes were obtained after infection of U-937 cells with promastigotes. Briefly, U-937 cells were dispensed in 24-well plates at a concentration of 300,000 cells/well and were treated with 1 µM of phorbol myristate acetate (PMA) (Sigma) for 48 h at 37 °C. Then, cells were infected with promastigotes in stationary growth phase (day 5) at a ratio of 1:15 cell/parasite and incubated for 3 h at 34 °C in 5% CO_2_. Cells were washed twice with PBS to eliminate not internalized parasites and fresh RPMI-1640 was added into each well (1 mL); plates were incubated again at 34 °C and 5% CO_2_ to allow intracellular differentiation of promastigotes to amastigotes form. After 24 h of infection, cells were ready to be used in antileishmanial testing assay as described below.

#### 4.3.3. *In Vitro* Cytotoxicity Using U-937 Cells

The cytotoxicity of compounds was assessed based on the viability of U-937 cells and evaluated by the MTT (3-(4,5-dimethylthiazol-2-yl)-2,5-diphenyltetrazolium bromide) method as described by others [26]. Briefly, into each well of a 96-well cell-culture dishes 100,000 cells/100 μL were dispensed in RPMI-1640 supplemented with 10% FBS and 100 μL of the corresponding concentrations of the corresponding product. Six two fold serial dilutions were were tested starting at 200 µg/mL (200, 100, 50, 25, 12.5 and 6.25 μg/mL). Cells were incubated at 37 °C with 5% CO_2_ for 72 h in the presence of compounds, and then the effect was determined by measuring the activity of the mitochondrial dehydrogenase by adding 10 µL/well of MTT solution (0.5 mg/mL) and incubating at 37 °C for 3 h. The reaction was stopped by adding 100 μL /well of 50% isopropanol solution with 10% sodium dodecyl sulfate for 30 min. Cell growth was determined based on the quantity of formazan produced, which was measured at 570 nm in a plate reader spectrophotometer (Bio-Rad, Hercules, CA, USA). Cells cultured in the absence of products were used as cell growth controls (negative control), while cells cultured in presence of amphotericin B (AmB, Sigma) and meglumine antimoniate (MA, Sanofi-Aventis, Bogotá, Colombia) were used as cytotoxicity controls (positive control). Each concentration was tested in triplicate in at least two independent experiments.

#### 4.3.4. *In Vitro* Leishmanicidal Activity on Intracellular Amastigotes

**Figure 2:**
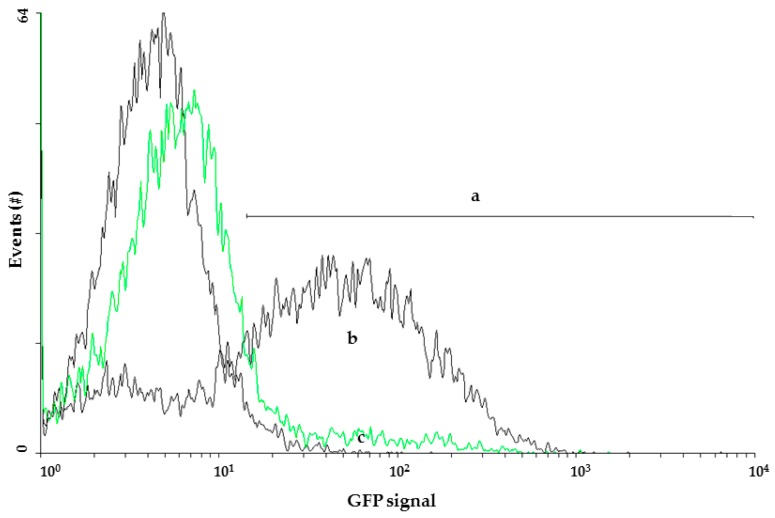
Flow cytometry analysis of GFP expressing parasites exposed to active and non-active *N*-(halomethylated) ammonium salts, non *N*-(halomethylated) ammonium salts and choline and halogenated cholines. The figure shows a representative fluorescence intensity signal from U-937 cells infected with *L. (V) panamensis*-pIR-eGFP exposed to any of the *N*-(halomethylated) ammonium salts, non *N*-(halomethylated) ammonium salts and choline and halogenated cholines compound. (**a**) shift of the relative increase in the average fluorescence for *L. (V) panamensis*-pIR-eGFP compared to plain parasites; (**b**) black histogram represents the signal produced by *Leishmania* exposed to non active compounds and; (**c**) green histogram represents the signal produced by parasites exposed to active compounds.

The effect of compounds against intracellular amastigotes of *L. (V) panamensis*-pIR-eGFP was evaluated by flow cytometry using the methodology described by others [27]. After 24 h of infection of U-937 cells, culture medium was replaced by fresh complete RPMI 1640 medium containing each compound at the corresponding concentration (four serial dilution were prepared starting at a concentration not exceeding the LC_50_ previously determined). Infected and treated cells were maintained at 34 °C and 5% CO_2_ for 72 h. After 72 h, cells were washed twice with 1 mL of cold PBS and cells were removed from the bottom plate with a trypsin/EDTA (250 mg/mL) solution. Recovered cells were centrifuged at 1100 rpm for 10 min at 4 °C, the supernatant was discarded and cells were washed with 1 mL of cold PBS and centrifuged at 1100 rpm for 10 min at 4 °C. Supernatant was discarded and cells were suspended in 500 μL of PBS and analyzed by flow cytometry (FC 500MPL, Cytomics, Brea, CA, USA) using histogram analyses (Figure 2). Infected cells treated with AmB or MA were used as control for antileishmanial activity (positive control) while infected cells incubated in culture RPMI 1640 medium alone were used as control for infection (negative control). Each concentration of compounds was tested in triplicate in two independent experiments.

#### 4.3.5. *In Vitro* Leishmanicidal Activity with Axenic Amastigotes

The ability of compounds to kill axenic amastigotes of *L. (V) panamensis* was determined based on the viability of the parasites after exposure to each compound evaluated by the MTT method following the methodology described by others [26]. Axenic amastigotes obtained as described above were harvested, washed, and adjusted at 2 × 10^6^ parasites/mL in fresh Schneider’s medium with 20% FBS. Into each well of a 96-well plate 100 µL of parasite suspension (2 × 10^5^ axenic amastigotes) were dispensed and then 100 µL of each concentration of the corresponding compound were added. Four serial dilutions were prepared starting at 100 µg/mL (100, 25, 6.25 and 1.56 μg/mL). Plates were incubated at 32 °C. After 72 h of incubation, the effect of each compound was determined by adding 10 µL/well of MTT and incubating at 32 °C for 3 h. The reaction was stopped as described above and the quantity of formazan produced was measured in a plate reader spectrophotometer (Bio-Rad) set at 570 nm. Axenic amastigotes cultivated in the absence of any compound but maintained under the same conditions were used as controls for growth and viability (negative control) while parasites cultivated in the presence of MA were used as controls for leishmanicidal activity (positive control). Assays were performed at least twice with three replicates per each concentration tested.

#### 4.3.6. Data Analysis

Cytotoxicity was determined according to the percentages of cell growth (viability) obtained for each tested compound or medium alone. Percentages of viability were calculated first using Equation (1):

% Viability = (O.D. of treated cells)/(O.D. of untreated cells) × 100(1)

where the O.D. of the untreated cells corresponds to 100% viability. Then, the percentage of cell growth inhibition was calculated using Equation (2):

% Cell growth inhibition = 100 − % Viability(2)

Both % viability and % cell growth inhibition were used to calculate the Lethal Concentration 50 (LC_50_) that corresponds to the concentration of drug that gives the half-maximal reduction of the cell growth using the Probit model [28]. The degree of cytotoxicity was established as convenient according to the LC_50_ values, using our own scale: high cytotoxicity: LC_50_ < 100 μg/mL, moderate cytotoxicity: 100 μg/mL < LC_50_ < 200 μg/mL; and potential non-cytotoxicity: LC_50_ > 200 μg/mL.

The antileishmanial activity in intracellular amastigotes was determined according to the reduction of infected cells and parasite load (viable parasites inside infected cells) obtained for each experimental condition. The percentage of infected cell inhibition and parasite load for each concentration of each compound were calculated first using Equation (3):

% infection = (% infected and treated cells ÷ % infected and untreated cells) × 100(3)

And then, the percentage of infection inhibition was calculated using Equation (4):

% Inhibition = 100 − % infection(4)

In turn, activity of compounds with axenic amastigotes was determined according to the reduction of parasite growth percentages obtained for each experimental condition. The percentage of parasite growth was calculated first using Equation (5):

% parasite growth = (O.D. treated ax. amastigotes)/(O.D. of untreated ax. amastigotes) × 100(5)

where the O.D. of the untreated axenic amastigotes corresponds to 100% parasite growth. Then, the percentage of parasite growth inhibition was calculated using Equation (6):

% parasite growth inhibition = 100 − % parasite growth(6)

The % of infection in U-937 cells and the % of inhibition of axenic amastigotes growth were used to calculate the Effective Concentration 50 (EC_50_) that corresponds to the concentration of drug that gives the half-maximal reduction of the infected cells and parasites using the Probit model [28]. The degree of leishmanicidal activity was also established as convenient according to the EC_50_ values, using our own scale: high leishmanicidal activity: EC_50_ < 25 μg/mL, moderate activity: 25 μg/mL < EC_50_ < 50 μg/mL; and potential non-activity: EC_50_ > 50 μg/mL.

The Selectivity Index (SI) was calculated by comparing the cytotoxicity and the leishmanicidal activity using Equation (7):

SI = LC_50_/EC_50_ (7)

## 5. Conclusions

Based on *in vitro* activity (EC_50_, μg/mL) against *L. (V) panamensis*, it was determined that the most active compound against *L. (V) panamensis* is compound **2c**, which has the longest carbon chain and a iodomethyl group on the nitrogen. When comparing the halomethylated salts **1** and **2** (series I) it is observed that their activity decreases with decreasing the carbon number of the salts that bear an iodomethyl group as a substituent, such that **2c** is more effective than **2b** and this, in turn, is more effective than **2a**. On the other hand, as a general trend, the salts bearing an iodomethyl group are more effective against *L. (V) panamensis* parasites than those bearing a chloromethyl group as a substituent. Currently, the structure-activity relationships via structural variations is being carried out.

## Figures and Tables

**Table 1:** Cytotoxicity against U-937 cells and antileishmanial activity of *N*-(halomethylated) ammonium salts, non *N*-(halomethylated) ammonium salts and choline and halogenated cholines.

Compound	Cytotoxicity	Intracellular Amastigotes		Axenic Amastigotes	
	LC_50_	EC_50_	SI	EC_50_	SI
**1a**	44.4 ± 4.9	38.4 ± 0.2	1.2	35.0 ± 5.7	1.3
**1b**	21.0 ± 2.0	46.8 ± 1.2	0.5	56.3 ± 3.7	0.4
**1c**	21.0 ± 0.5	49.4 ± 3.1	0.4	40.5 ± 3.3	0.5
**2a**	46.0 ± 5.4	27.8 ± 3.9	1.7	36.2 ± 0.1	1.3
**2b**	10.1 ± 0.2	**24.0 ± 0.7**	0.4	**19.7 ± 1.5**	0.5
**2c**	9.5 ± 0.3	**17.6 ± 1.0**	0.5	**14.0 ± 0.9**	0.7
**3a**	39.2 ± 4.8	30.0 ± 1.4	1.3	**25.0 ± 0.7**	1.5
**3b**	27.2 ± 0.7	**24.7 ± 1.9**	1.2	**25.0 ± 0.5 **	1.1
**3c**	12.5 ± 0.8	41.6 ± 2.8	0.3	34.1 ± 5.1	0.4
**4a**	17.9 ± 0.02	29.6 ± 2.8	0.6	**24.3 ± 1.3**	0.7
**4b**	17.8 ± 0.2	**23.9 ± 1.0**	0.7	**19.6 ± 1.8**	0.9
**4c**	372.0 ± 43.1	149.5 ± 17.9	2.5	47.2 ± 7.1	7.9
AmB	28.2 ± 0.5	**0.05 ± 0.001**	560	0.041 ± 0.001	690
MA	358.2 ± 1.7	**6.33 ± 0.86**	56.6	312.1 ± 18.6	1.4

Results reported as the mean value ± standard deviation of the half-maximum concentration in μg/mL. AmB: Amphotericin B; MA: meglumine antimoniate; the Selectivity Index (SI) = LC_50_/EC_50_. Bold data represent compounds with high activity against amastigotes of *L. (V) panamensis*.

## Abbreviations

The following abbreviations are used in this manuscript:

VL: Visceral leishmaniasis
ML: Mucocutaneous leishmaniasis
CL: Cutaneous leishmaniasis
MA: Meglumine antimoniate
AmB: Amphotericin B
EC_50_: Effective Concentration 50
LC_50_: Lethal Concentration 50
FBS: Fetal Bovine Serum
NNN: Novy-MacNeal-Nicholle
PMA: Phorbol 12-myristate 13-acetate
RPMI: Roswell Park Memorial Institute
O.D.: Optical Density
THF: Tetrahydrofuran
DMSO: Dimethylsulfoxide
